# Triangular Relationship between Sleep Spindle Activity, General Cognitive Ability and the Efficiency of Declarative Learning

**DOI:** 10.1371/journal.pone.0049561

**Published:** 2012-11-21

**Authors:** Caroline Lustenberger, Angelina Maric, Roland Dürr, Peter Achermann, Reto Huber

**Affiliations:** 1 Child Development Center, University Children's Hospital Zurich, Zurich, Switzerland; 2 Institute of Pharmacology and Toxicology, University of Zurich, Zurich, Switzerland; 3 Neuroscience Center Zurich (ZNZ), University and ETH Zurich, Zurich, Switzerland; 4 Zurich Center for Integrative Human Physiology (ZIHP) University of Zurich, Zurich, Switzerland; University of California Riverside, United States of America

## Abstract

EEG sleep spindle activity (SpA) during non-rapid eye movement (NREM) sleep has been reported to be associated with measures of intelligence and overnight performance improvements. The reticular nucleus of the thalamus is generating sleep spindles in interaction with thalamocortical connections. The same system enables efficient encoding and processing during wakefulness. Thus, we examined if the triangular relationship between SpA, measures of intelligence and declarative learning reflect the efficiency of the thalamocortical system. As expected, SpA was associated with general cognitive ability, e.g. information processing speed. SpA was also associated with learning efficiency, however, not with overnight performance improvement in a declarative memory task. SpA might therefore reflect the efficiency of the thalamocortical network and can be seen as a marker for learning during encoding in wakefulness, i.e. learning efficiency.

## Introduction

A beneficial effect of sleep for declarative memory has been discussed for a long time, but the extent of its impact or the underlying mechanism is still a matter of debate [1]. Sleep spindles, a major EEG characteristic of non-rapid eye movement (NREM) sleep, have been associated with such sleep dependent memory improvement [2]. Thus, learning of a declarative memory task resulted in an increase of sleep spindle density during subsequent sleep [3] and the sleep dependent performance improvement correlated with the number of sleep spindles [4]. However, there are also studies reporting no sleep dependent performance improvement in declarative memory tasks [e.g. 5], [6], [7]. The divergence regarding sleep dependent performance improvement could be due to differences in the task design [1]. For example, one major difference in the task design is the feedback for accuracy given in an immediate recall before sleep [8], [9]. Such a feedback might be associated with performance improvements in delayed recall after sleep [7].

Another interesting observation of sleep spindles is their relationship to measures of intelligence found in various studies [e.g. 10], [11], [12], [13]. This relationship fits with the finding that the topographic distribution of sleep spindle activity (SpA) might reflect functional anatomical differences between individuals [14].

How sleep spindles are generated may provide an explanation for the link between sleep spindles and measures of intelligence. Sleep spindles are the result of an interaction between the thalamic reticular nucleus (TRN) and thalamocortical connections [15], [16]. During wakefulness the TRN is a key component in a larger attentional network, enabling the thalamocortical system to encode and process relevant information more efficiently [for review see 17]. Efficient information processing is certainly an important factor for intelligence. Moreover, efficient attention allocation is also an important factor for learning efficiency [18].

In the present experiment we compared the performance improvement in a declarative memory task between an immediate and a delayed recall after sleep and examined their relationship to SpA and general cognitive abilities.

## Methods

The data of 15 male subjects were obtained from a larger study (Lustenberger et al., unpublished data). Subjects were paid for participation. The cantonal ethic commission in Zurich (Switzerland, study identification number: EK 2009-0120/2) approved the study and written informed consent was obtained from all participants.

All-night polysomnography was measured in the sleep laboratory for each subject during the experimental night. An adaptation night in the sleep laboratory was preceding every experimental night. Starting one week prior to the experimental night, the subjects were asked to maintain a regular sleep-wake schedule, verified by actigraphy and sleep diaries. Bedtime was adjusted to individual sleep habits, but the time in bed was held constant at eight hours. The subjects were all right-handed and in good general health. Subjects were excluded if they reported any sleep complaints, an irregular sleep-wake rhythm or showed low sleep efficiency. Other exclusion criteria were drug and medication abuse, smoking, a body mass index (BMI) lower than 19 or higher than 30 kg/m^2^, consumption of more than five alcoholic drinks per week, consumption of more than five caffeinated beverages or food items per day, learning disabilities and skin allergies. Only male participants participated in the study since the sleep spindle measures are known to be influenced by the menstrual cycle [19]. Subjects were aged between 18 and 20 years (19.3±0.8, mean ± SD).

The polysomnography included electroencephalogram (EEG), electroocculogram (EOG), electromyogram (EMG) and electrocardiogram (ECG). The electrodes for the EEG were placed according to standard configuration (10–20 system). The data were sampled at 1024 Hz with the polygraphic Amplifier Artisan (Micromed, Mogliano, Veneto, Italy). EEG electrodes were referenced to the vertex (Cz) and re-referenced to mastoids (A1, A2). The data were band-pass filtered between 0.5 Hz and 50 Hz. Sleep stage scoring of 20-second epochs was performed according to standard criteria (AASM Manual for Scoring Sleep) [20] with the software REMbrandt DataLab (Version 8.0; Embla Systems, Broomfield, CO, USA). Visual artefact exclusion was done during scoring on a four-second basis. Furthermore, semi-automatic artefact rejection was applied [21]. Fast Fourier transform (Referential derivation C4A1, Hanning window, average of five 4-s epochs, frequency resolution 0.25 Hz) was performed for EEG data during NREM sleep in order to assess SpA (spectral power in the 12–15 Hz frequency range).

The word pair task was used to assess sleep dependent performance improvement in declarative memory. 30 semantically unrelated word pairs were presented on a computer screen for six seconds each. After the presentation, there was an immediate recall, where the first words of the word pairs were presented in random order and the second one had to be recalled. To avoid primacy and recency effects, the first and last four word pairs of the presentation were excluded from recall. The subjects were asked to guess in case they did not remember the answer. No time limit for the answers was set and once the subjects entered their answer, feedback for accuracy was provided and the correct word pair was presented again for two seconds. The subjects were instructed to memorize the word pair again during feedback. In the morning, there was a delayed recall, where the procedure was the same as in the immediate recall. Performance improvement was defined as the difference in correct answers between delayed and immediate recall. Regarding the learning of new associations, two distinct parameters can be differentiated, which determine successful learning [22]. One aspect is the learning capacity, which is representing the maximal amount of information that can be stored. The other parameter is the learning efficiency, which determines how fast someone is able to learn until maximal capacity is reached. Thus, we assessed learning efficiency by the initial acquisition rate, which was calculated as the performance in the immediate recall, expressed as a percentage of the delayed recall performance. It measures how much of the individual learning capacity is already reached after the first presentation of the word pairs.

General cognitive ability was assessed by the number-joining test (German: Zahlen-Verbindungs-Test, ZVT) [23], measuring information processing speed (s^−1^). This measure is highly correlated with psychometric IQ measures and reflects the speed component of more extended intelligence tests [24].

15 additional participants were tested on the word pair task only. These subjects performed two recalls as the other subjects. However, the second recall was scheduled five minutes after the first (and did not include any sleep). The improvement from first to second recall was compared between the two groups in order to compare the amount of improvement after a five minute break and a whole night of sleep. Since no sleep measures were recorded in these additional subjects, inclusion and exclusion criteria were less strict. The 15 male subjects were aged between 19 and 23 years (21.4±0.9, mean ± SD), in good general health and had no learning disabilities.

15 subjects from the first experiment that successfully performed the word pair task were included in the analysis. Sleep dependent performance improvement, initial acquisition rate, SpA, information processing speed and performance improvement in the group with no sleep were normally distributed (Kolmogorov-Smirnov all *P*>0.05). Thus, we used parametric tests. Statistical analysis was performed with the program PASW Statistics 18 (SPSS, version 18.0.0).

## Results and Discussion

In agreement with other studies [e.g. 9], [25], [26], [27], in subjects having a night of sleep in between recalls, we found a significant performance improvement in the number of correctly recalled word pairs from immediate to delayed recall (+4.6±1.9, paired t-test, *P*<0.05). There was no ceiling effect observable in both recalls. Thus, no subject was able to recall all word pairs correctly.

**Figure 1:**
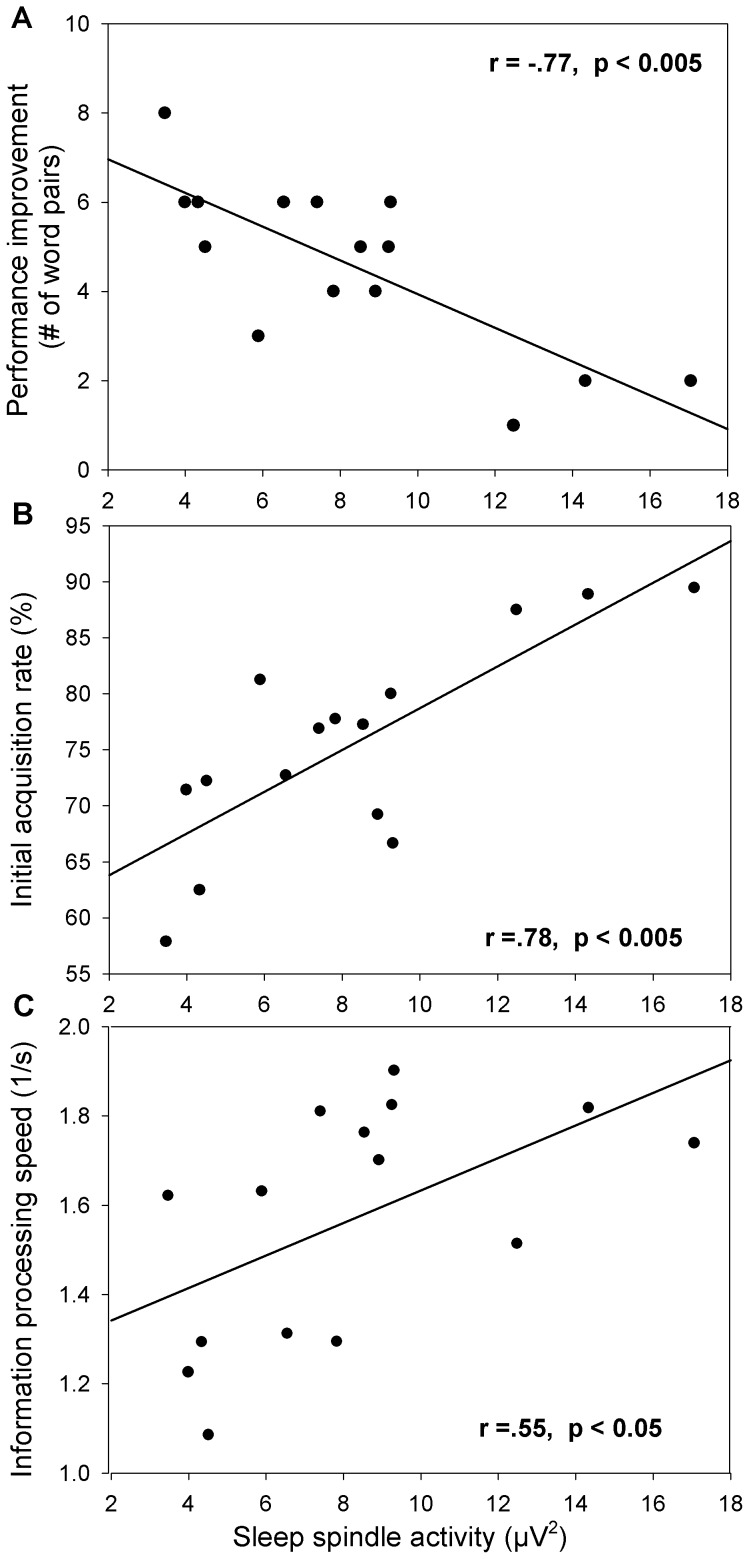
The triangular relationship of sleep spindle activity, performance improvement and initial acquisition rate. Correlation between NREM sleep spindle activity (EEG power in 12–15 Hz range) and **A** performance improvement (delayed minus immediate recall), **B** initial acquisition rate (immediate in percentage of delayed recall), **C** information processing speed (number-joining test).

Pearson correlation coefficients were calculated to assess if the performance improvement after sleep is associated with SpA. There was a negative correlation between SpA and performance improvement explaining about 60% of the variance (figure 1A). Thus, the more SpA a subject displayed the smaller the improvement in performance the next day. This observation is in contrast to previous observations of a positive relationship between sleep spindles and sleep dependent performance improvement [4], [7], [26]. What might such a discrepancy explain?

In a first step we assessed whether the performance improvement between immediate and delayed recall is sleep dependent. To do so, we compared the performance improvement between the group with sleep between recalls and the performance improvement of the group with five minutes of waking between recalls. Our results showed that also the group with a five minute break exhibited a significant performance improvement (+7.1±3.3, paired t-test, *P*<0.001). The performance improvement was significantly higher in the five minutes wake compared to the sleep group (unpaired t-test; *P*<0.05). Hence, performance improvement was not primarily dependent on sleep but may have resulted from the feedback given during immediate recall. However this finding does not exclude the possibility of a preserving effect of sleep on memory. As seen in other studies, forgetting during wakefulness seems to occur to a higher degree as compared to during sleep (e.g. [28]). Thus the larger improvement in the waking control group might be explained by less forgetting in between recalls compared to the sleep group.

Nevertheless, the question remains what the negative correlation between sleep SpA and performance improvement might mean.

In order to address this question, we introduced the initial acquisition rate as the inverse of the performance improvement since in the present study this performance improvement is not sleep dependent.

**Figure 2:**
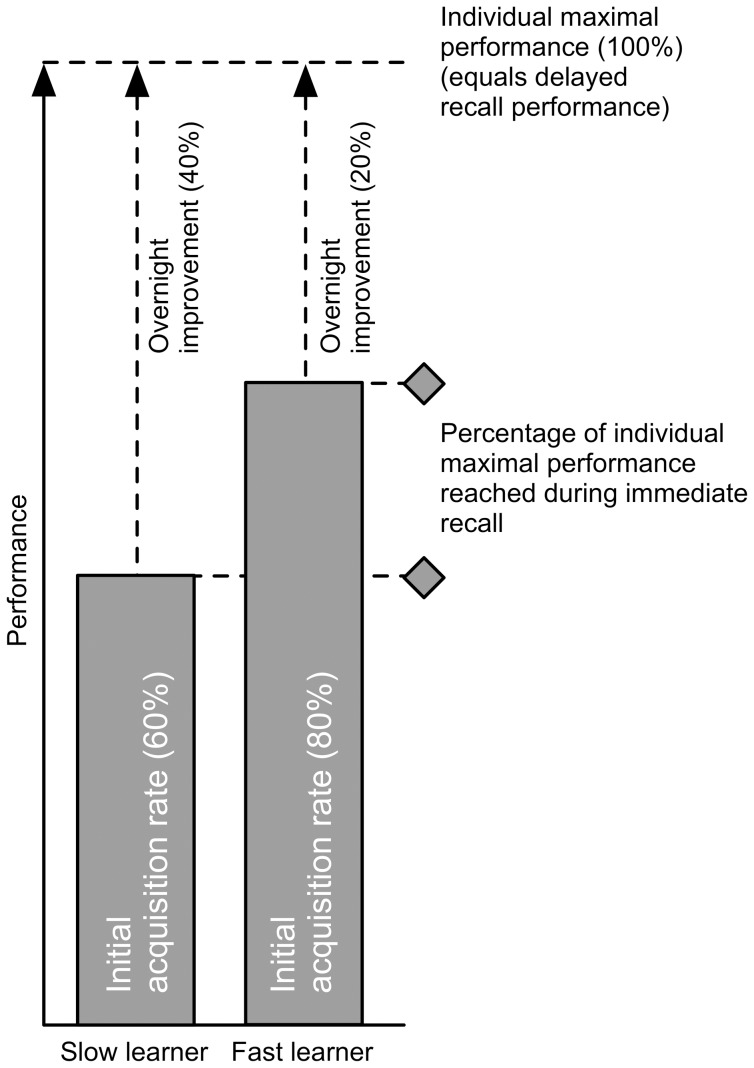
Schematic view of the inverse relationship between the overnight performance improvement and the initial acquisition rate. The initial acquisition rate reflects how much of the individual maximal performance is already reached during the immediate recall. A slow learner has a lower initial acquisition rate (e.g. 60%) compared to a fast learner (e.g. 80%). This means that a slow learner reaches less of his maximal capacity in the course of the first learning session (acquisition phase) and may benefit more from a second presentation of the word pairs (during feedback). Thus, his overnight performance improvement may be larger (e.g. 40%). Vice versa, a fast learner is able to get closer to his maximal capacity during the first presentation of the learning material and may have less overnight performance improvement (e.g. 20%). Note that delayed recall performance is reflecting the maximal capacity an individual has ( = 100%).

Thus, a faster learner would reach much of its maximal capacity during the first presentation of stimuli (in the evening) and would less improve after additional presentation of the word pairs (the next day). As expected we found a positive correlation between SpA and the initial acquisition rate, our measure of learning efficiency, explaining approximately 60% of the variance (figure 1B). In other words, more efficient learning is associated with higher SpA. This finding explains the negative correlation between overnight performance improvement and SpA since more efficient learning corresponds to less overnight performance improvement in the present experiment (figure 2).

The performance improvement as well as the initial acquisition rate is highly correlated with SpA. However, we can not conclude statistically which of the two variables is more adequate to describe the relationship between declarative learning and sleep spindles. Nevertheless, several points favor the initial acquisition rate as a key factor: 1) the negative correlation between overnight performance improvement and SpA is in contrast to results reported in the literature [4], [7], [26], 2) the negative correlation does not fit any rational interpretation, and 3) the improvement is mainly resulting from feedback given during the recall and therefore not primarily sleep dependent. Hence, we concluded that the relationship of declarative learning and sleep spindles is reflected by the association of SpA with learning efficiency, rather than with overnight performance improvement.

Finally, we assessed the relationship between SpA and general cognitive ability (information processing speed). In agreement with the literature [e.g. 10], [11], [12], [13], we found a positive correlation between SpA and information processing speed (average information processing speed, 1.6±0.3 s^−1^; figure 1C).

The positive relationship between sleep spindles and general cognitive ability shows that sleep spindle measures may indeed be a reflection of anatomical and functional differences in the thalamocortical system [14]. Furthermore, the positive association between SpA and learning efficiency supports the view that sleep spindles may reflect the efficiency of the thalamocortical system, i.e. myelinisation of thalamocortical fibers, number and strength of thalamocortical connections [13], [29]. Therefore, the results imply that subjects with higher SpA have a more efficient thalamocortical system, which is associated with higher general cognitive abilities and enables the subjects to encode and process information more efficiently. It has already been reported that reaction times in a selective attention task are negatively correlated with sleep spindles [30], which is in agreement with the present findings. The observation that SpA is strongly related to learning efficiency might also explain some of the discrepancies observed in other studies about sleep dependent performance improvement using a word pair task. Subjects with high SpA have a more efficient processing and therefore may be able to strengthen memories better in the first place. Since these individuals preserve more of the information until the delayed recall, the impression may arise that sleep spindles are causing consolidation. Our results rather indicate that sleep spindles reflect learning as it occurs during encoding in wakefulness. This interpretation is supported by the fact that only subjects, which showed a strong acquisition of the memory content during training showed sleep dependent performance improvement [31]. Furthermore, an increase in the number of sleep spindles by neurofeedback training was not accompanied by increased sleep dependent performance improvement, but rather resulted in superior performance in immediate recall [6]. These findings might be interpreted as follows: neurofeedback training results in increased efficiency of the thalamocortical system, e.g. the strengthening of thalamocortical connections. This increased efficiency might lead to an enhancement of encoding performance and processing speed. An alteration of intrinsic pacemaker properties, i.e. of the thalamus, is indeed likely to occur as a result of neurofeedback [for review see 32]. Moreover, Rauchs et al. [33] found a correlation between sleep spindles and performance only in the immediate recall in subjects suffering from Alzheimer's disease (AD). Interestingly, patients suffering from AD showed reduced sleep spindles. Furthermore, they have not found any sleep dependent performance improvement. The authors mention that this might be due to deficient encoding of the material during learning. Matzel et al. [34] support this view by reporting that age-related memory impairments are mostly due to deficient learning and not due to faster forgetting or deficient retrieval. Beyond that, also healthy aged subjects have reduced selection efficiency [35] and show decreased SpA, even though less pronounced than in AD [33].

A link between encoding efficiency and sleep spindle measures is also supported by the deficits in early encoding in patients suffering from Schizophrenia [e.g. 36], [37] and the fact, that sleep spindles are dramatically decreased in this population [38]. Ferrarelli et al. [39] conclude that the reduction of SpA in Schizophrenia is likely the result of deficient corticothalamic connections, leading to an impaired sleep spindle generating system. Furthermore, Keshavan et al. [40] report that scores of attention and psychomotor speed are negatively correlated with SpA in Schizophrenic patients.

The performance improvement in the present experiment was shown to be at least partly resulting from the feedback given during immediate recall, since recall was similar after sleep and after five min of wakefulness. This finding might be different in word pair tasks using semantically related word pairs, since learning of semantically related and unrelated word pairs have been shown to entail different mechanisms of learning as for example reflected in dissimilar event related potential (ERP) measures [41].

Even though the mean age is significantly increased in the waking control group, age seems not to confound our results, since we found no correlation between age and the performance improvement after 5 minutes (p>0.9), neither after sleep (p>0.6).

In the present experiment we were able to show that SpA (of a single night recording) is positively associated with learning efficiency. However, further experiments are needed to examine the relationship between changes of SpA from a baseline without learning and learning efficiency, since sleep spindle measures are altered after intense learning [3], [7].

## Conclusion

Sleep spindles are reflecting the efficiency of the thalamocortical system, which is the source of sleep spindles and is important for successful encoding and processing during wakefulness. An efficient system is reflected by higher SpA on the one hand, and enables faster learning on the other hand. It is also reflected in higher general cognitive abilities. Thus, measuring SpA during sleep might be a promising method to draw conclusions about the efficiency of the thalamocortical system, cognitive and encoding abilities in healthy and clinical populations. Moreover, sleep spindle measures are presumably useful to investigate the thalamocortical system and how it reacts to learning or cognitive training. A successful manipulation of the thalamocortical system therefore would be an important treatment approach in subjects with impaired cognitive abilities and with deficits in thalamoreticular or thalamocortical circuits as for instance in schizophrenic subjects [38], [39].
